# Religious ceremonies and the ethical development of medical sciences students: A qualitative study on participation barriers and perceived value

**DOI:** 10.1371/journal.pone.0350651

**Published:** 2026-06-04

**Authors:** Amir Hossin Moradpour Dehnavi, Abolfazl Alavi, Amin Beigzadeh, Ali Reza Yusefi

**Affiliations:** 1 Sirjan School of Medical Sciences, Sirjan, Iran; 2 Department of Islamic Studies, Sirjan University of Technology, Sirjan, Iran; 3 Education Development Center, Sirjan School of Medical Sciences, Sirjan, Iran; 4 Student Research Committee, Sirjan School of Medical Sciences, Sirjan, Iran; Shandong University, CHINA

## Abstract

Religious ceremonies can play a pivotal role in shaping ethical values among medical sciences students. However, participation in such ceremonies is often influenced by multiple academic, social, and cultural factors. This study aimed to explore the perceived value of religious ceremonies and the barriers affecting student participation in these practices within the context of their ethical development. This qualitative study was conducted at Sirjan School of Medical Sciences in southern Iran from March to July 2025, using a latent content analysis approach grounded in the interpretivist paradigm. Semi-structured, in-depth interviews were conducted with 33 students from diverse academic programs and backgrounds. Data were analyzed inductively based on Graneheim and Lundman’s framework using MAXQDA 2022 software. Trustworthiness was ensured through Lincoln and Guba’s criteria including credibility, confirmability, dependability, and transferability. Seven main themes and twenty- four subthemes emerged. The themes included: (1) Time and Academic Pressure (e.g., course overload, exam clashes); (2) Perceived Irrelevance (e.g., disconnection from professional goals); (3) Cultural and Personal Beliefs (e.g., secular upbringing, concerns about religious imposition); (4) Social Dynamics (e.g., fear of judgment, peer influence); (5) Institutional Support (e.g., lack of promotion, insufficient facilities); (6) Perceived Ethical Value (e.g., development of professionalism and compassion); and (7) Emotional and Community Benefits (e.g., stress relief, sense of belonging, spiritual recharge). While religious ceremonies hold perceived ethical and emotional value for many students, numerous academic, institutional, and cultural barriers limit participation. Integrating religious practices into educational contexts in a more inclusive, flexible, and voluntary manner could enhance students’ moral development without alienating diverse beliefs.

## Introduction

The ethical development of medical sciences students is a cornerstone of professional healthcare education, as future healthcare providers are expected to demonstrate not only clinical competence but also moral integrity, empathy, and accountability in their practice [1].

While traditional medical ethics education has primarily relied on formal curricula, increasing attention has been paid to the role of informal and hidden curricula – such as cultural, religious, and spiritual experiences – in shaping ethical attitudes and behaviors [2]. Among these, religious ceremonies represent a potentially influential yet underexplored dimension of students’ ethical socialization, particularly in culturally and religiously rooted societies such as Iran [3].

Religious ceremonies are collective rituals that embody moral narratives and promote reflection on human suffering, responsibility, and justice [4]. These elements are closely aligned with the moral values expected of healthcare professionals [5]. Despite this theoretical alignment, students’ engagement with such ceremonies is variable, and existing evidence suggests that multiple barriers – including academic pressure, time constraints, and changing belief systems – may limit participation [6,7].

In the Iranian context, where religion is deeply embedded in social and cultural life, the potential role of religious ceremonies in fostering ethical awareness among medical sciences students remains insufficiently explored [8]. Although settings such as Sirjan School of Medical Sciences provide regular exposure to such rituals, there is limited empirical understanding of how students perceive their ethical value or why participation may be declining.

Previous studies in Iran and other Muslim-majority countries have examined the relationship between religiosity and moral outcomes such as empathy or moral reasoning [9,10]. However, these studies are predominantly quantitative and focus on individual beliefs rather than collective religious practices, failing to capture the lived experiences and contextual meanings associated with participation in religious ceremonies. Importantly, there is a lack of qualitative evidence explaining how and why medical sciences students engage with (or disengage from) religious ceremonies and how they perceive their ethical value. This gap highlights the need for qualitative inquiry to explore how medical sciences students interpret the ethical significance of religious ceremonies and what factors shape their engagement or disengagement. Therefore, the present study aims to explore both the perceived ethical value of religious ceremonies and the barriers to student participation, using a qualitative approach grounded in students’ lived experiences. By focusing specifically on this underexplored intersection between collective religious practices and ethical development, this study seeks to provide context-sensitive insights that can inform ethics education and professional identity formation in healthcare settings.

In sum, this qualitative study seeks to explore the following key questions: [1] What are the main barriers that prevent medical sciences students from participating in religious ceremonies during their education? and [2] How do students perceive the potential ethical value of these ceremonies for their personal and professional development in the healthcare field?

## Methods

### Study design and setting

This study employed a qualitative research design using a latent content analysis approach to explore the perceived value of religious ceremonies and the barriers to participation among students of Sirjan School of Medical Sciences in southern Iran. The study was conducted between March 13^th^ and July 15^th^, 2025 and was grounded in the interpretivist paradigm, which is well-suited for examining subjective experiences and social phenomena [11].

The decision to use qualitative methodology was driven by the study’s aim to gain an in-depth understanding of the underlying beliefs, social influences, and institutional factors shaping students’ engagement or disengagement with religious practices within the broader context of their ethical development in academic and clinical settings. In line with the interpretivist stance, the research questions were designed to explore meaning-making processes rather than to test predefined hypotheses, allowing participants to articulate their subjective interpretations in their own terms.

### Philosophical paradigm and researcher positioning

This study was grounded in the interpretivist paradigm, which is based on the ontological assumption that reality is multiple, subjective, and socially constructed, and the epistemological view that knowledge is co-created through interaction between the researcher and participants. This paradigm is particularly suitable for exploring complex, meaning-laden phenomena such as students’ perceptions of religious ceremonies and their ethical significance, as it allows for in-depth understanding of lived experiences within their cultural and social contexts. From this perspective, the researchers were not considered detached observers but active participants in the knowledge production process. Their role as instruments of data collection and interpretation required ongoing reflexivity and sensitivity to participants’ narratives. During interviews, efforts were made to create a dialogical and non-judgmental environment that facilitated open sharing of experiences, acknowledging that meanings were co-constructed through these interactions. Furthermore, the interpretivist stance informed the analytical approach by emphasizing inductive coding, contextual interpretation, and the identification of latent meanings rather than merely surface-level descriptions. The researchers continuously reflected on how their own backgrounds and assumptions might influence data interpretation, and took deliberate steps – such as team discussions and reflexive note-taking – to ensure that findings remained grounded in participants’ perspectives.

Importantly, the research team included members with diverse academic backgrounds, including medical education and Islamic studies, which made reflexivity and critical self-awareness essential throughout the research process. Particular attention was paid to managing potential influence of disciplinary perspectives during data interpretation.

### Participants

Participants were selected from a wide range of academic programs offered at the school, including medicine, nursing, midwifery, public health, laboratory sciences, and allied health disciplines. The study sought to ensure variation in terms of gender, field of study, academic year, and self-reported levels of religious engagement, thereby enabling the exploration of diverse perspectives. A total of 33 students participated in the study through semi-structured, in-depth interviews.

### Inclusion and exclusion criteria

The inclusion criteria required participants to be currently enrolled in one of the academic programs offered by Sirjan School of Medical Sciences across various medical sciences disciplines, to express willingness to participate voluntarily, and to have the ability to articulate their views and experiences related to religious ceremony participation. Individuals who withdrew during the interview process or declined to provide informed consent were excluded from the study.

### Sampling and recruitment strategy

A purposive sampling strategy with maximum variation was employed to capture a broad spectrum of perspectives. Participants were initially identified through coordination with faculty members and student representatives across different departments, who facilitated access to students with diverse academic backgrounds and varying levels of religious engagement. In addition, recruitment announcements were disseminated via student social media groups and institutional communication channels, inviting interested students to voluntarily contact the research team. To ensure inclusion of participants with differing degrees of religious involvement, initial screening questions were used to assess self-reported frequency of participation in religious ceremonies (e.g., regular, occasional, or non-participation). Furthermore, a snowball sampling technique was applied, whereby interviewed participants were asked to introduce peers with differing viewpoints or experiences. Potential participants were approached either in person or through direct messaging, provided with detailed information about the study objectives, and invited to participate. Recruitment continued until data saturation was achieved.

### Pilot testing of the interview guide

Prior to commencing formal data collection, the interview guide was pilot tested with three medical sciences students to ensure the clarity, cultural appropriateness, and relevance of the questions. Based on their feedback, minor revisions were made to the wording and sequence of questions to enhance comprehension and flow. The finalized interview guide began with a broad, open-ended question inviting participants to reflect on their experiences of attending or not attending congregational prayers during their student life. This was followed by probing questions organized around seven broad sensitizing domains designed to guide exploration of the topic rather than to structure analysis. These domains included: time and academic pressure, relevance to professional goals, cultural and personal beliefs, social dynamics, institutional support, perceived ethical value, and emotional and community benefits. Importantly, these domains were used only to ensure comprehensive coverage of relevant aspects during interviews and did not function as predefined analytical categories. Probing and follow-up questions were employed throughout the interviews to encourage depth and elaboration, such as “Can you explain more?”, “Do you have an example?”, or “How did that affect you?” (see supporting information).

Importantly, the data obtained from pilot interviews were used solely to refine the interview guide and were not included in the final analysis.

### Data collection

All interviews were conducted face-to-face in a quiet and private setting within the school premises. The interviews were carried out by two trained and experienced qualitative researchers (ARY and AHMD), both of whom had prior experience in conducting in-depth interviews and qualitative fieldwork. Interviews lasted approximately 30–45 minutes and were conducted in Persian, the participants’ native language, to facilitate a natural and comfortable conversation. Each interview was audio-recorded with the participant’s informed consent. In addition to audio recordings, the researchers also took field notes to capture non-verbal cues and contextual details that could enrich the interpretation of data. Interviews were transcribed verbatim on the same day, and transcripts were cross-checked against the audio recordings to ensure accuracy and completeness. Anonymity and confidentiality were strictly maintained throughout the study, and all transcripts were de-identified using participant codes.

Data collection continued until thematic saturation was reached, defined as the point at which no new codes or categories emerged in three consecutive interviews, as confirmed through regular team discussions and iterative data analysis.

### Data analysis

Data analysis was guided by the principles of latent content analysis, following the methodological framework proposed by Graneheim and Lundman [12]. The researchers initially immersed themselves in the data by reading transcripts multiple times to achieve a holistic understanding of participants’ narratives. Meaning units relevant to the research questions were then identified within the text, condensed while preserving their core meaning, and subsequently coded inductively. These codes were continuously compared for similarities and differences and grouped into subcategories, which were then abstracted into broader categories and overarching themes that captured the latent meanings underlying participants’ experiences. Initial coding was conducted independently by two researchers, who coded the transcripts line-by-line. The independently generated codes were then compared in iterative meetings to enhance consistency and interpretive rigor. Disagreements in coding or interpretation were resolved through discussion and, when necessary, consultation with a third senior qualitative researcher to reach consensus. To ensure transparency of the analytic process, the transformation from raw data to final themes was carefully documented. For instance, the participant statement “I barely have time for sleep, let alone attending a religious event. Between lectures, hospital rounds, and assignments, my day is fully packed” (P03) was first identified as a meaning unit reflecting time-related constraints. This was then condensed into the meaning “lack of time due to heavy academic and clinical workload prevents participation in religious ceremonies”, and subsequently coded as academic overload limits participation in non-academic activities. This code was grouped under the subcategory heavy course load, which ultimately contributed to the broader theme time and academic pressure. This analytic progression illustrates how raw narrative data were systematically abstracted into higher-order conceptual categories while preserving contextual meaning. Throughout the analysis, an analytic audit trail was maintained, including coding manuals, category development records, decision logs, and reflexive memos documenting interpretive decisions. These materials enabled continuous tracking of analytical decisions from raw data through to final thematic development. The entire analytical process was facilitated using MAXQDA software (version 2022), which supported systematic organization, retrieval, and comparison of data segments.

### Trustworthiness

To ensure the rigor and trustworthiness of the study, the criteria established by Lincoln and Guba – namely credibility, dependability, confirmability, and transferability – were applied [13]. Credibility was strengthened through prolonged engagement with participants across the data collection period, as well as member checking with a subset of participants selected to represent different academic disciplines, academic years, and levels of religious engagement. During member checking, preliminary interpretations and summarized meanings were presented to these participants either immediately at the end of interviews or through follow-up contact. Participants generally confirmed the accuracy of interpretations, and minor clarifications (e.g., rephrasing of motivational statements) were incorporated into the final analysis. In addition, peer debriefing sessions were conducted biweekly with two qualitative research experts outside the immediate research team. These sessions focused on reviewing emerging codes and themes, challenging interpretations, and discussing alternative explanations. Feedback from these discussions contributed to refining category boundaries and improving analytical consistency. Dependability was addressed by documenting the analytical process in detail and maintaining consistency in data handling. An explicit audit trail was maintained, including raw interview transcripts, coding manuals, category development tables, decision logs, and reflexive memos, which documented all major analytical decisions and revisions throughout the research process. These documents allow external researchers to trace the full analytical pathway from raw data to final themes. Confirmability was ensured by maintaining a transparent audit trail of decisions made during coding and theme development, including reflexive notes and analytic memos. This was further strengthened through continuous reflexive engagement by the research team, ensuring that findings were grounded in participants’ narratives rather than researchers’ preconceptions or disciplinary perspectives. Transferability was enhanced by providing thick descriptions of the research context, participant characteristics, and analytical interpretations. Specifically, Sirjan School of Medical Sciences is located in a culturally and religiously embedded region of southern Iran where religious ceremonies are socially visible and integrated into community life. At the same time, the school hosts a diverse student population from various provinces of Iran. Participants represented multiple disciplines (medicine, nursing, midwifery, public health, laboratory sciences, and allied health), different academic years, and varied living conditions (dormitory and non-dormitory students), enhancing the contextual richness and applicability of the findings to similar settings.

### Researcher reflexivity

Given the sensitive nature of religious beliefs and ethical development, the research team engaged in reflexive practices throughout the study. The primary researchers had academic backgrounds in medical education and qualitative research and were familiar with the cultural and religious context of the participants. Importantly, one member of the research team had an academic affiliation with the Department of Islamic Studies, which necessitated heightened reflexivity regarding how disciplinary perspectives might influence the research process, particularly in relation to religious meanings and interpretations.

To minimize potential bias, the researchers acknowledged their preconceptions regarding the value of religious practices and actively reflected on these assumptions during data collection and analysis. In practice, this process was framed as reflexive awareness rather than strict bracketing, meaning that the researchers did not attempt to fully suspend their perspectives, but instead continuously examined and documented how these perspectives could shape interpretation. This approach is consistent with the interpretivist paradigm, which recognizes the co-constructed nature of meaning.

The research team also reflected on how their positionality could influence the research process. Specifically, the presence of a researcher with Islamic Studies expertise may have shaped participants’ perceptions during interviews, potentially increasing expectations of religious alignment or influencing the way questions were interpreted. To mitigate this, interviews were conducted in a neutral, non-judgmental manner, emphasizing voluntary participation and academic rather than ideological interest in the topic.

Reflexive notes were maintained throughout the study to document personal reflections, assumptions, and decision-making processes. Regular team discussions were conducted to critically examine interpretations and ensure that findings were grounded in participants’ narratives rather than researchers’ prior beliefs. These discussions explicitly focused on identifying instances where disciplinary perspectives could have influenced coding decisions, and alternative interpretations were actively considered to reduce interpretive bias.

### Ethics approval and consent to participate

This study is approved by the Sirjan School of Medical Sciences Ethics Committee under ID number IR.SIRUMS.REC.1403.052. The study protocol was also registered in the school research system (project code: 403000040). All methods were carried out in accordance with relevant guidelines and regulations. All participants provided written informed consent and were assured of their right to withdraw from the study at any point without any consequences.

## Results

A total of 33 students from Sirjan School of Medical Sciences participated in this study. Most participants (42.42%) were aged 18–20. The majority were enrolled in nursing programs (24.24%). Most were female (69.70%), single (84.85%), lived in dormitories (72.73%), and were not employed (81.82%). Detailed demographic characteristics of the participants are presented in Table 1.

**Table 1:** Demographic characteristics of participants.

Characteristic	Category	Frequency (n)	Precentage (%)
Field of Study	Medicine	5	15.15
	Nursing	8	24.24
	Midwifery	3	9.09
	Public Health	2	6.06
	Environmental Health	3	9.09
	Occupational Health	3	9.09
	Anesthesia	2	6.06
	Laboratory Sciences	4	12.12
	Emergency Medicine	2	6.06
	Medical Technologies	1	3.03
Age	18-20	14	42.42
	21-24	11	33.33
	25-28	4	12.12
	29-32	4	12.12
Gender	Female	23	69.70
	Male	10	30.30
Marital Status	Single	28	84.85
	Married	5	15.15
Place of Residence	Dormitory	24	72.73
	Non-Dormitory	9	27.27
Employment Status	Employed	6	18.18
	Unemployed	27	81.82

**Fig 1:**
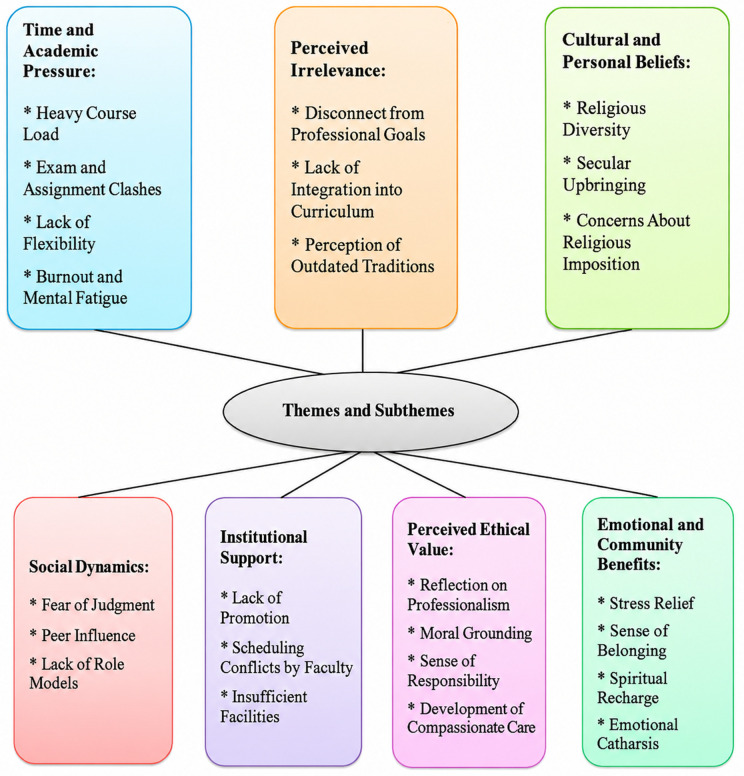
Thematic map of participation barriers and ethical value of religious ceremonies among medical sciences students.

This qualitative study identified seven main themes and twenty- four sub-themes related to barriers preventing medical sciences students from participating in religious ceremonies and their perceptions of the ceremonies’ ethical value. The main themes include time and academic pressure, perceived irrelevance to professional goals, cultural and personal beliefs, social dynamics, institutional support, perceived ethical value, and emotional and community benefits. Each theme encompasses several sub-themes that capture the detailed experiences and attitudes of the students. The findings highlight a complex balance between external constraints and internal motivations, suggesting that while students face significant challenges to participation, many still recognize important ethical and emotional benefits from engaging in religious ceremonies. The themes and sub-themes with illustrative student quotations are summarized in Table 2 and Fig 1.

**Table 2:** Thematic structure of barriers to participation and perceived ethical value of religious ceremonies among medical sciences students.

Main Theme	Subtheme Illustrative	Participant Quotation	Code
**Time and Academic Pressure**	Heavy Course Load	*I barely have time for sleep, let alone attending a religious event. Between lectures, hospital rounds, and assignments, my day is fully packed. Even weekends are used to catch up on studies or rest.*	*P03*
	Exam and Assignment Clashes	*Ceremonies often happen right before midterms or deadlines. It’s hard to justify going when I have so much academic pressure and fear of underperforming in exams.*	*P14*
	Lack of Flexibility	*There’s no formal option to make up for missed classes or tasks due to attending these events. So we’re forced to choose academics over everything else—even meaningful experiences.*	*P18*
	Burnout and Mental Fatigue	*By the end of the day, I’m mentally exhausted. Even if I have the time, I just don’t have the energy to engage in something reflective or spiritual.*	*P30*
**Perceived Irrelevance**	Disconnect from Professional Goals	*My goal is to be a competent doctor. I don’t immediately see how attending a religious event supports that. It feels more personal than professional.*	*P11*
	Lack of Integration into Curriculum	*If ethics or values from ceremonies were linked to course objectives, maybe I’d care more. Right now, it feels like a completely separate world.*	*P22*
	Perception of Outdated Traditions	*Some parts of the ceremonies feel outdated or too formal. I wish they spoke more to today’s challenges in healthcare instead of repeating old scripts.*	*P06*
**Cultural and Personal Beliefs**	Religious Diversity	*These events often represent a specific religious tradition. While I respect that, I sometimes feel like I don’t belong because my beliefs differ or aren’t reflected.*	*P09*
	Secular Upbringing	*I grew up in a non-religious family, so these practices are unfamiliar to me. It’s not that I’m against them—I just don’t relate to them the way others might.*	*P07*
	Concerns About Religious Imposition	*Even when attendance is optional, it can feel like a subtle pressure—like being moral means being religious. That can be uncomfortable for someone with different views.*	*P16*
**Social Dynamics**	Fear of Judgment	*Sometimes I want to attend, but I’m afraid my classmates will see me differently—like I’m trying to be holier-than-thou. I don’t want that kind of attention.*	*P25*
	Peer Influence	*If my friends or roommates skip these events, I usually do too. You don’t want to be the only one doing something different in such a close-knit group.*	*P19*
	Lack of Role Models	*When senior students and respected professors never attend, it gives the impression that these ceremonies aren’t relevant or valuable in the long run.*	*P01*
**Institutional Support**	Lack of Promotion	*I often find out about these events too late—through word of mouth or an unnoticed flyer. It feels like they’re not really prioritized by the university.*	*P13*
	Scheduling Conflicts by Faculty	*No one reschedules lectures or exams for these ceremonies. So even if you want to go, you have to miss something important, which discourages attendance.*	*P04*
	Insufficient Facilities	*Sometimes the ceremonies happen in small, crowded places that don’t feel appropriate. That affects the whole experience and makes it feel less meaningful.*	*P21*
**Perceived Ethical Value**	Reflection on Professionalism	*Listening to the values shared in ceremonies—like compassion, humility, and service—reminds me of what kind of doctor I want to be, beyond grades and technical skills.*	*P10*
	Moral Grounding	*These events help me reconnect with the reasons I entered medicine. They offer ethical reminders that stay with me long after I return to the classroom.*	*P27*
	Sense of Responsibility	*After participating, I feel more grounded in my obligations—not only to patients, but also to society and the moral expectations of being a healthcare provider.*	*P05*
	Development of Compassionate Care	*The stories and prayers shared during these ceremonies help me imagine myself as a more empathetic, human-centered clinician, especially in moments of patient suffering.*	*P15*
**Emotional and Community Benefits**	Stress Relief	*These ceremonies give me a rare opportunity to slow down, breathe, and reflect. It’s like a mental detox from the academic pressure and emotional fatigue.*	*P20*
	Sense of Belonging	*Gathering in one space for a shared purpose creates a sense of unity that I don’t usually feel in daily student life. It makes me feel less alone*	*P08*
	Spiritual Recharge	*Even if I’m not deeply religious, I find comfort in the quiet moments. It restores my inner peace and reminds me of bigger meanings behind what we do.*	*P12*
	Emotional Catharsis	*Sometimes I get emotional during these ceremonies, and it feels like a release. It helps me process everything I’ve been holding in during the semester.*	*P17*

### Variations across participant characteristics

Although the primary aim of this study was not to conduct subgroup comparisons, several notable patterns emerged across participant characteristics. Students in clinical disciplines, particularly medicine and nursing, more frequently emphasized time constraints, academic workload, and burnout as primary barriers, reflecting the demanding nature of clinical training. In contrast, students in earlier academic stages and non-clinical fields (e.g., public health and laboratory sciences) more often highlighted social influences and uncertainty regarding the relevance of religious ceremonies. Additionally, nursing and midwifery students tended to express stronger appreciation for the emotional and ethical value of participation, particularly in relation to compassionate care and patient-centered practice. Regarding gender, while both male and female participants shared similar structural concerns, female students more frequently articulated the emotional and community-related benefits, such as sense of belonging and emotional catharsis. These observations, although exploratory, provide additional depth to the findings and suggest potential directions for future research.

## Discussion

This qualitative study aimed to explore the barriers to participation in religious ceremonies and their perceived ethical and emotional value among medical sciences students. Thematic analysis revealed a complex interplay between structural, personal, cultural, and institutional factors influencing students’ engagement, along with meaningful insights into the moral and emotional benefits derived from such activities. Importantly, the findings also revealed internal tensions between perceived ethical value and lived constraints, suggesting that students simultaneously experience attraction toward and disengagement from religious ceremonies within the same educational environment. These findings contribute to a nuanced understanding of how spiritual practices intersect with professional identity formation in the context of modern healthcare education.

Time and Academic Pressure emerged as one of the most prominent barriers. Participants frequently referred to a heavy course load, exam and assignment clashes, and lack of flexibility in scheduling as major deterrents to attending religious events. These findings echo those of Almasry et al. (2017), who found that the increasing academic workload in medical schools significantly limits student participation in non-curricular ethical enrichment programs [14]. Similarly, Wachholtz and Rogoff (2013) reported that rigid exam schedules and burnout were major contributors to disengagement from spiritually enriching activities [15]. The burnout and mental fatigue described by participants further align with Dyrbye et al. (2016), who linked chronic academic stress with reduced motivation to engage in reflective or moral practices [16]. From a theoretical perspective, this pattern can be interpreted through the lens of the Theory of Planned Behavior, where perceived behavioral control (lack of time and energy) significantly constrains intention even when attitudes toward participation are positive. These observations suggest that ethical development is often deprioritized in high-pressure academic environments, despite its long-term value in clinical decision-making and empathy-based care.

The theme of Perceived Irrelevance reflects a deeper structural issue in curriculum design. Many students noted a disconnect between religious ceremonies and professional goals, arguing that spiritual programs were not adequately linked to their future roles as healthcare providers. This perception mirrors the findings of Guck and Kavan (2006), who observed that many medical students see spirituality as peripheral rather than integral to professional formation [17]. Moreover, the lack of integration into the curriculum reinforces previous critiques by Puchalski et al. (2014), who called for more holistic and spiritually inclusive medical education [18]. This disconnect can be further understood through Professional Identity Formation theory, which emphasizes that students prioritize experiences they perceive as directly contributing to their emerging clinical identity. When religious ceremonies are not explicitly framed as relevant to professional competencies, they are constructed as peripheral rather than formative experiences. The perception of outdated traditions among some participants suggests a generational and cultural shift, where younger students may struggle to see the relevance of traditional religious formats unless they are contemporized or contextualized. Ray and Wyatt (2018) also noted that when spiritual practices are presented without modernization or dialogue, they risk being dismissed by students as obsolete or irrelevant [19].

Cultural and Personal Beliefs played a pivotal role in shaping students’ attitudes toward participation. Students from religiously diverse or secular upbringings expressed feelings of discomfort or detachment, a finding consistent with Rockenbach and Mayhew (2024), who emphasized that faith-based programs in pluralistic societies must be inclusive and voluntary to avoid alienating minority voices [20]. Concerns about religious imposition were particularly salient; some students feared that attending religious events could be perceived as forced conformity. This aligns with the observations of Verbree et al. (2023), who discussed the tension between institutional values and personal beliefs in multicultural academic settings [21]. These findings can be interpreted through Social Identity Theory, where individuals evaluate participation in group-based rituals in relation to their in-group identity. When religious ceremonies are perceived as representing a dominant cultural identity, students from minority belief systems may experience symbolic exclusion, even in voluntary settings. These findings underscore the importance of fostering an environment that respects belief diversity while still offering ethically meaningful experiences.

The influence of Social Dynamics was another critical factor. Students reported fear of judgment by peers, especially in contexts where religious participation was viewed as “too traditional” or “unfashionable.” This echoes Edwards et al. (2024) study on peer conformity, where students hesitated to express moral or spiritual values for fear of ridicule [22]. Moreover, the lack of visible role models who actively engaged in religious or ethical reflection created a vacuum of leadership. Mohammadi et al. (2021) emphasized that moral modeling by students and faculty significantly enhances the transmission of ethical values [23]. Peer influence was not uniformly negative; in some cases, students followed the group norm to avoid social isolation, whether or not they valued the ceremonies. This duality highlights how professional identity formation occurs within peer cultures that simultaneously enable and constrain ethical expression.

The theme of Institutional Support revealed systemic shortcomings. Students mentioned lack of promotion of religious events, scheduling conflicts created by faculty, and inadequate physical spaces for religious gatherings. These concerns mirror those reported by Taverna et al. (2019), who argued that logistical barriers and administrative neglect often marginalize ethical and spiritual education [24]. Lucchetti et al, (2012) similarly noted that when institutions do not invest in ethical spaces, student participation and perception of value significantly decline [25]. These findings suggest that institutional culture communicates implicit value signals; when ethical or spiritual activities are not structurally supported, students interpret them as low-priority components of the hidden curriculum.

Despite these challenges, students strongly endorsed the Perceived Ethical Value of religious ceremonies. Participants described these events as moments for reflection on professionalism, moral grounding, and the development of a sense of responsibility. This is consistent with Koenig (2012), who highlighted the role of religious participation in fostering moral reflection and accountability among healthcare trainees [26]. These ceremonies, even when brief or symbolic, seemed to help students connect their clinical duties with deeper ethical commitments, highlighting a central tension in the data whereby ethical awareness does not automatically translate into ethical action.

Finally, Emotional and Community Benefits were emphasized across diverse participant backgrounds. Students described religious gatherings as opportunities for stress relief, emotional catharsis, and spiritual recharge. Similarly, Pargament et al. (2000) emphasize that religious coping mechanisms, including participation in communal rituals, provide significant emotional support and facilitate coping with life’s challenges [27]. These emotional benefits appear to complement, rather than compete with, the ethical outcomes of such participation; however, this also reveals a critical tension, as students experience meaningful emotional and existential gains, yet these benefits are often insufficient to outweigh the structural and social constraints that limit actual participation.

In sum, the findings highlight a persistent paradox in ethical development, where students value religious ceremonies as morally meaningful spaces, yet systemic pressures, identity negotiations, and peer norms significantly restrict actual engagement. This tension suggests that ethical formation in medical education is not only a cognitive or value-based process, but also a socially negotiated and structurally constrained phenomenon. At the same time, the results indicate both the potential of religious ceremonies to foster ethical growth and the multifaceted barriers that limit student participation, underscoring the need for an integrative approach that bridges academic, social, cultural, and institutional dimensions. When designed inclusively and promoted appropriately, religious ceremonies can serve as transformative experiences in the ethical development of future healthcare professionals.

## Conclusion

This study highlights the complex interplay between personal beliefs, academic demands, institutional structures, and social dynamics in shaping medical students’ participation in religious ceremonies. While numerous barriers hindered engagement—including time pressure, cultural diversity, and perceived irrelevance—students also recognized profound ethical and emotional benefits from participation, including enhanced moral reflection, greater compassion, and a sense of community belonging. These findings underscore the potential of religious and spiritual activities to contribute meaningfully to the ethical development of future healthcare professionals, provided they are presented in an inclusive, flexible, and contextually relevant manner.

### Policy implications and recommendations

The findings of this study call for academic institutions, particularly medical universities, to re-evaluate the integration of spiritual and ethical programming within their educational environments. Rather than offering generic optional ethical sessions, universities could implement structured “ethical reflection modules” embedded within specific clinical rotations (e.g., pediatrics, emergency medicine, palliative care), where students engage in facilitated group reflection on real clinical experiences related to suffering, compassion, and responsibility. These sessions should be designed as 20–30 minute structured debriefings facilitated by trained faculty members, using culturally resonant narratives – including but not limited to religious stories, patient narratives, and ethical dilemmas – to stimulate reflection without imposing any specific worldview. Institutional support in the form of scheduling flexibility, adequate facilities, and visible promotion of such activities can significantly improve engagement. Faculty development programs should train educators in facilitating reflective discussions rather than delivering didactic moral content. In addition, evaluation mechanisms such as reflective journals or structured feedback forms could be used to assess the impact of these interventions on students’ ethical reasoning and professional identity development over time. Promoting inclusivity and respect for diverse beliefs must remain central to any policy reform to ensure participation is empowering rather than alienating.

### Limitation

While this qualitative study offers rich insights, it is not without limitations. The sample was limited to students from a single cultural and academic setting, which may restrict the transferability of findings to broader populations. Additionally, participants’ self-selection may have introduced bias, as those with stronger opinions – positive or negative – about religious ceremonies may have been more inclined to participate. The sensitive nature of the topic may also have led some students to withhold or temper their true views during interviews, despite assurances of confidentiality.

### Suggestions for future research

Future studies could adopt comparative or longitudinal designs to examine how perceptions and participation in religious or ethical activities evolve over time and across different academic disciplines or cultural contexts. Quantitative studies could help validate the themes identified here and measure the impact of spiritual engagement on specific professional outcomes, such as empathy, ethical reasoning, or burnout resilience. It would also be valuable to explore faculty and institutional perspectives on the role of religion and spirituality in ethical development to create more holistic and responsive educational strategies.

## Supporting information

S1 File Semi-structured interview guide used for data collection, provided in both Persian and English. (DOCX)
