# Supplementary material for: Religious ceremonies and the ethical development of medical sciences students: A qualitative study on participation barriers and perceived value
Source: PLoS One. 2026 Jun 4;21(6):e0350651. doi: 10.1371/journal.pone.0350651 (PMC13235870; doi:10.1371/journal.pone.0350651)
Supplement: S1 File — (DOCX) [file pone.0350651.s001.docx]

# Interview Guide: Religious Ceremonies and the Ethical Development of Medical Sciences Students: A Qualitative Study on Participation Barriers and Perceived Value

Part 1: English Version

Semi-Structured Interview Guide

**Opening Question:**

Please describe your experience of attending or not attending religious ceremonies during your student life.

**Domain 1: Time and Academic Pressure**

How does your academic workload influence your participation in these ceremonies?

Have you experienced conflicts between exams, assignments, and religious events?

How do fatigue and mental exhaustion affect your decision to attend?

**Domain 2: Relevance to Professional Goals**

In your opinion, how are these ceremonies related to your future professional role (e.g., as a doctor or nurse)?

Do you perceive any connection between these ceremonies and your academic training?

**Domain 3: Cultural and Personal Beliefs**

How do your personal beliefs or family background shape your views on these ceremonies?

Have you ever felt that these ceremonies do not align with your beliefs? Please explain.

**Domain 4: Social Dynamics**

What role do peers and the social environment play in your decision to attend or not attend?

Does fear of judgment or social perception influence your behavior?

**Domain 5: Institutional Support**

To what extent does the university support or promote these ceremonies?

How do you evaluate the facilities and organization of these events?

**Domain 6: Perceived Ethical Value**

Has participation in these ceremonies influenced your ethical or professional values?

Do these ceremonies help you better understand concepts such as responsibility, empathy, or commitment?

**Domain 7: Emotional and Community Benefits**

Do these ceremonies help reduce stress or improve your emotional well-being?

Do they create a sense of belonging or social connection?

**Probing Questions:**

Can you elaborate more?

Can you give an example?

How did that affect you?

Part 2: Persian Version

**راهنمای مصاحبه نیمه‌ساختاریافته**

***سؤال آغازین (کلی)***

لطفاً درباره تجربه خود از شرکت یا عدم شرکت در مراسم‌های مذهبی در دوران دانشجویی توضیح دهید.

***حوزه 1: فشارهای زمانی و تحصیلی***

برنامه درسی و حجم دروس شما چه تأثیری بر شرکت در این مراسم‌ها دارد؟

آیا تا به حال تداخل زمانی بین امتحانات یا تکالیف و این مراسم‌ها را تجربه کرده‌اید؟

از نظر انرژی ذهنی و خستگی، شرایط شما چگونه بر تصمیم‌گیری‌تان اثر می‌گذارد؟

***حوزه 2: ارتباط با اهداف حرفه‌ای***

به نظر شما این مراسم‌ها چه ارتباطی با آینده حرفه‌ای شما (مثلاً پزشک یا پرستار شدن) دارند؟

آیا احساس می‌کنید محتوای این مراسم‌ها با آموزش‌های دانشگاهی شما مرتبط است؟ چرا؟

***حوزه 3: باورهای فرهنگی و فردی***

باورهای شخصی یا پیشینه خانوادگی شما چه تأثیری بر نگرش شما نسبت به این مراسم‌ها دارد؟

آیا تا به حال احساس کرده‌اید که این مراسم‌ها با باورهای شما هم‌خوانی ندارند؟ لطفاً توضیح دهید.

***حوزه 4: تعاملات اجتماعی***

نقش دوستان، همکلاسی‌ها یا محیط اجتماعی در تصمیم شما برای شرکت یا عدم شرکت چیست؟

آیا نگرانی از قضاوت دیگران بر رفتار شما تأثیر گذاشته است؟

***حوزه 5: حمایت نهادی***

دانشگاه تا چه حد از برگزاری یا اطلاع‌رسانی این مراسم‌ها حمایت می‌کند؟

آیا امکانات و شرایط برگزاری را مناسب می‌دانید؟

***حوزه 6: ارزش‌های اخلاقی ادراک‌شده***

آیا شرکت در این مراسم‌ها بر نگرش اخلاقی یا حرفه‌ای شما تأثیر داشته است؟

آیا این مراسم‌ها به شما در درک مفاهیمی مانند مسئولیت‌پذیری، همدلی یا تعهد کمک کرده‌اند؟

***حوزه 7: مزایای عاطفی و اجتماعی***

آیا این مراسم‌ها تأثیری بر کاهش استرس یا بهبود حال روحی شما داشته‌اند؟

آیا شرکت در این مراسم‌ها حس تعلق یا ارتباط اجتماعی در شما ایجاد می‌کند؟

***سؤالات پیگیری:***

می‌توانید بیشتر توضیح دهید؟

مثال خاصی دارید؟

این موضوع چه تأثیری بر شما داشت؟
